# Incidence and determinants of neonatal mortality in the first three days of delivery in northwestern Ethiopia: a prospective cohort study

**DOI:** 10.1186/s12884-021-04122-8

**Published:** 2021-09-23

**Authors:** Mulugeta Dile Worke, Afework Tadele Mekonnen, Simachew Kassa Limenh

**Affiliations:** 1 Department of Midwifery, Debre Tabor University, Debre Tabor, Ethiopia; 2 Population and Family Health, Jimma University, Jimma, Ethiopia; 3 Department of Midwifery, Bahir Dar University, Bahir Dar, Ethiopia

**Keywords:** Neonatal death, Follow-up, Amhara region, Ethiopia

## Abstract

**Background:**

Addressing sustainable development goals to reduce neonatal mortality remains a global challenge, and it is a concern in Ethiopia. As a result, the goal of this study was to assess the incidence and determinants of neonatal mortality in the first 3 days among babies delivered in the referral hospitals of the Amhara National Regional State.

**Methods:**

A hospital-based prospective cohort study was conducted among 810 neonates in the first 3 days of delivery between March 1 and August 30, 2018. The neonates were followed up from the time of admission to 72 h. Interviewer-administered questionnaires and medical record reviews were conducted for data collection. Data were entered into Epi-data manager version 4.4 and analysed using STATA™ version 16.0. The neonate’s survival time was calculated using the Cox-Proportional hazards model.

**Results:**

The overall incidence of neonatal mortality in this study was 151/1000 births. Neonatal mortality was significantly higher among neonates whose mothers came between 17 and 28 weeks of gestation for the first visit; among those whose mothers labour was not monitored with a partograph, mothers experienced postpartum haemorrhage and developed a fistula first 24 h, and experienced obstructed labour. However, 39% were less risky among neonates whose mothers were directly admitted and whose mothers had visited health facilities in less than 1-h, both.

**Conclusions:**

This study revealed that approximately 1 in 7 neonates died within the first 3 days of life. The determinants were the timing of the first antenatal visit, quality of labour monitoring, maternal complications, and delay in seeking care. Thus, scaling up evidence-based interventions and harmonising efforts to improve antenatal care quality, promote institutional deliveries, provide optimal essential and emergency obstetric care, and ensure immediate postnatal care may improve neonatal survival.

**Supplementary Information:**

The online version contains supplementary material available at 10.1186/s12884-021-04122-8.

## Background

The newborn period is critical in the miracle of life and death. In 2018, the global neonatal mortality rate was estimated to be 18 deaths per 1000 live births, with 28 deaths per 1000 live births in Sub-Saharan Africa, and in 2016, it was 30 deaths per 1000 live births in Ethiopia [1, 2]. According to a systematic review in developing countries, despite more than 99% of neonatal deaths in low and middle-income countries, these settings lack overall or cause-specific neonatal deaths [3]. The same systematic review also revealed that 57% of neonates died in the first 3 days of life, and two-thirds of these deaths occurred within the first 24 h. Furthermore, this review indicated that among 52% of under-five deaths in the Southeast Asia region, one-third of the neonatal mortality occurred within the first 3 days of life. Among 30% of the under-five deaths in the World Health Organization (WHO) African region, 17% of deaths occur within the first day of life [3]. The WHO also verifies that the danger of death is most significant in the first 24 h of life, with more than half of all neonatal deaths occurring within the first week of life [4]. In Ethiopia, a study conducted at a referral specialised teaching hospital revealed that the death of babies aged 1–3 days was riskier than 4–7 days [5].

In low and middle-income countries, it has been suggested that the scaling-up of evidence-based interventions that begin during the antenatal period, coordinated efforts aimed at improving the quality of antenatal care, promoting institutional deliveries, providing optimal essential and emergency obstetric care, and ensuring immediate postnatal care of neonates are essential [3, 6]. Consequently, some countries, including Ethiopia, have substantially improved targeted coverage for some interventions. However, these coverages have not resulted in the expected magnitude of reduction in neonatal mortality rate, specifically neonatal mortality within the first 3 days of life. The lack of a concurrent rise in the coverage of essential interventions in the continuum of care could be one of the possible reasons [3]. For example, a recent study in Ethiopia indicated that only 12.1% of women completed the continuum of maternal care services, and 25.1% did not receive any care during their recent births [7].

Although the perinatal mortality rate definition starts at 28 weeks of gestation in low and middle-income countries (LMICs), the mortality rate is higher than the developed countries that stated the definition from 20 weeks of gestation. The perinatal mortality rate includes all stillbirths and neonatal deaths in a given period over the total number of births multiplied by thousands [8]. In LMICs, there is a lower preterm newborns’ survival rate, and the numerator for perinatal mortality rate includes all fetal deaths with a gestational age of 28 weeks and above and all neonatal deaths within 7 days of life [8]. However, according to a global network study, in LMIC registries, most neonatal deaths occurred in babies > 37 weeks of gestation, weighing at least 2500 g shortly after birth [9]. Previous studies have also identified multiple risk factors for perinatal mortality rate in LMICs [10–14]. Poverty contributes to many newborn fatalities, either by increasing risk factors such as maternal infection or limiting access to sufficient care [15]. Studies have also revealed that the proportions of common causes of perinatal mortality rate include prematurity (17%); asphyxia (25%); infection (37%); tetanus (7%); diarrhoea (3%); congenital malformations (4%); and other causes (7%) [16–19]. Almost all the studies were conducted at the community level and within 28 days of life and recommended prevention through better medical care and hospitalisation in the intrapartum and early neonatal period [9].

Neonatal mortality rates and determinants in the early neonatal period (the first 3 days of life in this case) are essential for arranging programs and identifying suitable interventions. Local and recent evidence about the spread of the problem could help implement programmatically relevant decision-making [10]. However, despite a high number of deaths in the first 3 days, data are scarce in the first 3days of life, particularly at the tertiary level of care. Moreover, since both biology and empirical data suggest that the cause of death distribution differs substantially between these periods, separate cause-of-death estimates are required for the first 3 days of life, within the first 7 days, and within 28 days of life [10]. Therefore, this study aimed to address the incidence and determinants of newborn death in the first 3 days of life among babies delivered in referral hospitals.

## Methods

### Study setting and period

This study was conducted at the maternity wards of referral hospitals in the Amhara Regional State between March 1 and August 30, 2018. The National Regional State is located between 9° 20′ and 14° 20′ North latitude and 36° 20′ and 40° 20′ East longitude in the Northwestern part of Ethiopia. According to the Central Statistics Agency, the projected total population estimate Amhara Region in 2020/21 is 22,536,999 (11,236,853 males and 11 300,146 females). Of these, 20.08% were urban residents [20]. According to the annual performance report published by the Federal Ministry of Health of Ethiopia in the 2009 Fiscal Year, the region had 68 hospitals, 841 health centres, and 3342 health posts [21]. Dessie, Felege-Hiwot, University of Gondar, Debre-Birhan, and Debre-Markos were referral hospitals at the time of data collection for this study. Each referral hospital was expected to serve a population of 5 million people. The University of Gondar Teaching Referral Hospital (UoGH), Felege Hiwot Referral Hospital (FHH), and Debre Markos Referral Hospital (DMH) were selected for this study.

The UoGH serves the residents of Gondar town and the neighbouring zones. The NICU was established 20 years ago and serves as the region’s tertiary referral unit, caring for high-risk newborns born at the hospital, referrals from other health facilities and home deliveries. Outpatient clinics, emergency departments, paediatrics and malnutrition wards, and neonatal intensive care units (NICUs) were among the services provided by paediatric and child health departments for rural and urban populations. Although neonatal hospitalisation varies seasonally, the annual average admission rate is 1140. There was no mechanical ventilators or continuous positive airway pressure (CPAP) equipment in this 32-bed NICU, including radiant heaters and nine incubators.

On the other hand, Bubble CPAP was locally developed for neonates with respiratory distress syndrome (RDS). There were also four incubators and three phototherapy machines for term babies. The babies were given oxygen via nasal prongs or a nasal catheter connected to oxygen cylinders or concentrators. Ampicillin and gentamicin were the most usually prescribed antibiotics for sepsis treatment. Medications were given through a peripheral vein, with the umbilical vein being used on a few occasions. The NICU has four rooms, which were helpful for preterm and term babies, infectious diseases, and maternity, where relatively stable neonates and newborns require kangaroo mother care. Seven medical interns, two pediatric residents, one paediatrician, and 17 nurses staff the NICU [22].

Felege Hiwot referral Hospital was a teaching hospital for Bahir Dar University and served the Bahir Dar special zone, west Gojjam zone, Awi zone, South Gondar Zone. More than 7 million people were living in these zones. The FHH was established in 1963 and has been in operation since then. Medical, surgical, gynaecological, orthopaedic, intensive care units, paediatrics, and ophthalmological wards with 375 beds and 561 employees currently provide health care services. Approximately 6300 neonates are diagnosed with various health issues each year. There were 60 beds, five paediatricians, and 20 nurses in the neonatal ward [23].

Furthermore, the DMH hospital served as a teaching hospital for Debre Markos University was the only referral hospital found in the East Gojjam Zone. This hospital serves a population of over 3.5 million individuals within its catchment area. For extremely ill neonates and those who require neonatal care, the hospital also offers neonatal intensive care. There were 27 nurses, one paediatrician, and two general practitioners working in the NICU. Ten NICU beds, four kangaroo mother care beds, 19 mother side beds, eight radiant warmers, and six incubators were available in the unit. The regular nursing procedures in the NICU were phototherapy, umbilical transfusion, oxygen administration, nasogastric tube insertion, intravenous infusion, urinary catheterisation, lumbar puncture, and CPAP. In 2017, this hospital provided neonatal intensive care services for 1419 neonates [24].

### Study design and population

An institution-based prospective cohort study was conducted among a cohort of term pregnant mothers and newborns admitted to three systematically selected referral hospitals. All term pregnant mothers (≥37 weeks gestational age (GA)) were admitted to the selected referral hospitals included in this study. Additionally, neonates discharged with an appointment and normal status were followed using mothers’ phones and the nearby health extension workers till 3 days of life. Then, they followed up until they gave birth, and their neonates were followed up for a total of 72 h. Cohorts of newborns who were delivered from women aged 15–49 years were included. Those born to women with mental illnesses who could not hear or speak due to their disease and twins were excluded from the study.

### Sample size and sampling technique

The sample size of 832 was calculated using Epi-info version 7 stat calc software. The following assumptions of the incidence ratio of early neonatal death of 369 per 2142 deliveries [25], 95% confidence level, the margin of error 2.75, and 15% lost follow-up.

Systematic random sampling was used to identify 832 admitted term pregnant women enrolled in the follow-up study. First, a simple random sampling (i.e., lottery method) technique selected the three hospitals. The study subjects were then allocated the proportion of the expected admitted number of term pregnant women per referral hospital, 300 each for UoGH and FHH, and 210 for DMH. The computed sample was then chosen in order from each referral hospital.

### Variables

Times-to-event, the event of interest was early neonatal death and dichotomised as (alive =1 and died = 0). The determinant variables included socio-demographic and economic factors: ethnicity, religion, place of residence, marital status, education status of the mother, and occupational status of the mother, age of mother, maternal and neonatal related factors: ANC follow up, parity gravidity, mode of delivery gestational age, birth weight, age of neonate at discharge, and sex of neonate. Neonatal illnesses include respiratory distress, perinatal asphyxia, sepsis, congenital malformation, hyaline membrane disease, and meconium aspiration syndrome—care/ service-related factors: Partograph follow-up, length of stay, and obstetric complications.

### Data quality assurance and questionnaire

First, we prepared the English questionnaire, translated it to Amharic’s local language, and back to English by different individuals to check its consistency. The survey was pretested on 42 mothers (14 exposed and 28 unexposed cohorts) in Debre Tabor Hospital, which differs from the study hospitals. The questionnaire (Additional file 1) was then assessed for its clarity and completeness. Some skip patterns were corrected, and questions difficult to ask were rephrased. The questionnaire had three parts. The first part was socio-demographic factors (i.e., maternal age, body mass index, age at first marriage, age at early pregnancy, age at first delivery, ethnicity, residence, marital status, educational status, husband educational status, occupation, an estimated distance of home from health institution, determining range from primary health institute to referral hospital, religion, and income). The reproductive factors constitute the second part, like gravidity, parity, gestational age, referral status, birth attendant, previous cesarean section, mode of final delivery, antenatal care attendance, number of ANC visits, duration of labour before the presentation, prior history of abortion, and obstetric complications. The third part was that programmatic factors included infrastructure and transportations. The completed questionnaires were checked day-to-day for inclusiveness, correctness, clarity, and consistency by the supervisors and the principal investigators, and necessary corrections and changes were made. During data entry and analysis, complete and consistent variables were checked using frequency distributions, cross-tabulations, sorting in ascending, and descending order.

### Data collection process

Because of the day and night allocation of data collectors in each hospital, six bachelor (two per hospital) holders experienced midwives collected data interchangeably (by shift), and three general practitioners (1 per each hospital) were supervised the process. Three days of training were given based on the study’s objective and the value of collecting the actual data. The structured questionnaire was discussed in detail, going through every question, and clarification was provided. A field manual was prepared for the supervisors and data collectors for use during data collection. The six data collectors were present in the respective hospitals during the complete 24 h.

The data collectors collected the data daily. Medical record review and an interviewer-administered questionnaire were used to collect data on the intended variables of interest until the time of discharge from the hospital or 72 h. We retained only the previously collected data were retained for analysis in cases of readmission. We assessed the neonates for the entire period of hospital stay (both in the maternity ward and neonatal intensive care unit). There was no follow-up after hospital discharge.

### Data management and analysis

Early neonatal mortality was the event of interest, coded as “1” for failure and “0” for censored. Time-to-event was considered by subtracting the date of admission from the time of the event. Data entered into Epi-Data manager version 4.4 and analysed using STATA™ version 16.0. for the follow-up time and age of the cohort, we calculated the mean and standard deviation. The cox-Proportional hazard model was used to determine risk factors for newborns’ survival time delivered at the hospitals. A tolerance level with a cut point of 0.2 was used to omit multicollinearity.

The Kaplan Meier curves were used to estimate survival time. The log-rank test was used to look at statistical variances between the groups of variables. Statistical significance was declared at *p*-value < 0.05. A summary statistic of proportions, including hazard ratio and 95% confidence intervals, was used. Screening of risk factors for the newborn’s death employed bivariate Cox regression for each variable one at a period. Those variables with *p* < 0.25 throughout the bivariate Cox regression analysis were taken as an entrant variable to control possible confounders for the multivariate Cox regression model.

### Operational definitions

**Early neonatal mortality:** This refers to a neonate’s death within the first 72 h of life.

**Neonatal survival:** is referred to as being alive until the end of the follow-up period (72 h).

**Term pregnancy:** It is defined as a pregnancy lasting between 37 and 42 weeks of gestation.

**Maternal First Delay** refers to the delay after the onset of actual labour to reach a health facility.

## Results

During the 72 h’ observation, 810 newborns (97.4% of the sample) followed for 37,454 new-borns-hours at the three hospitals. The mean (+SD) length of stay at the four hospitals was 46.23(+ 29.31) hours. Of the 810 newborns, 10.4% were stillbirths, 17.1% were alive but complicated, and 72.5% were alive without complications.

### Maternal and neonatal clinical characteristics

Out of the 84 stillbirths, 25 were from primiparous women, 15 deaths in the first 72 h out of the 38 deaths observed among women who gave 2 to 4 births. As Table 1 shows, 81.9% of the newborns who survived were mothers who received antenatal care. Out of 84 stillbirths, 58 of them were diagnosed with intrauterine fetal death before starting actual labour, whereas the rest 26 were fresh stillbirths during delivery (Table 1).

**Table 1:** New-borns and maternal clinical characteristics of Amhara regional state referral hospitals, Northern Ethiopia, 2018

Variables	Categories	Stillbirth	Neonates died in the first 72 h	Neonatal Survived
		Frequency(%)	Frequency(%)	Frequency(%)
Parity	One child	25(3.1)	13(1.6)	292(36.1)
	2 to 4 children	33(4.1)	15(1.8)	292(36.1)
	5 children and above	26(3.2)	10(1.2)	104(12.8)
Received Antenatal care	Yes	71(8.8)	35(4.3)	664(81.9)
	No	13(1.6)	3(.4)	24(2.9)
Place of delivery	Health institution	68(8.4)	31(3.8)	599(74.1)
	Home	15(1.8)	7(0.9)	88(10.9)
Newborn birth weight	< 2500	52(6.2)	31(3.8)	544(67.2)
	> = 2500	32(3.9)	7(.8)	144(17.8)
APGAR score of the newborn	0–3	69(8.5)	7(0.8)	0(.0)
	4–6	2(0.3)	6(.7)	32(3.9)
	7–10	13(1.6)	25(3.1)	656(80.9)
Fetal outcome in the uterus	IUFD	58(7.2)	0(.0)	0(.0)
	NRFHR	14(1.7)	18(2.2)	184(22.7)
	RFHR	12(1.5)	20(2.5)	504(62.2)
Mode of delivery	Vaginal	33(4.1)	18(2.2)	340(41.9)
	Ceserean section (C/S)	51(6.3)	20(2.5)	348(42.9)

*IUFD* intra-uterine fetal death, *NRFH* non-reassuring fetal heart rate, *RHFR* Reassuring fetal heart rate

### Neonatal illness

**Fig. 1:**
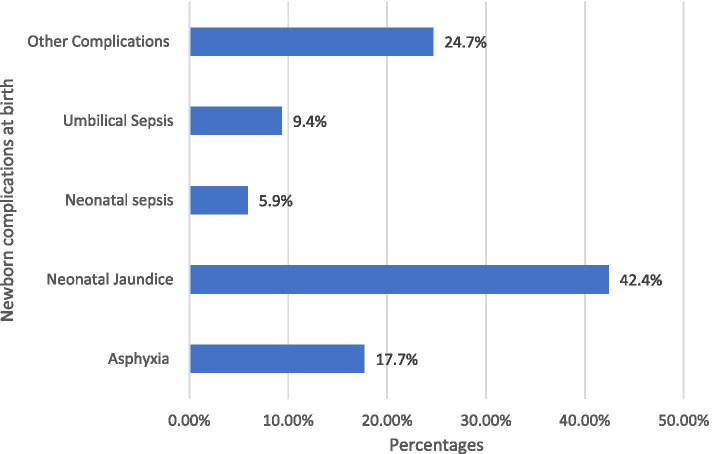
Causes of Neonatal morbidity in the first three days of delivery in Amhara regional state referral hospitals, northwestern Ethiopia

The leading cause of disease in Amhara regional state referral hospitals was neonatal jaundice 52(42.4%), followed by other complications 30(24.6%) like birth trauma, congenital anomalies, and asphyxia 22(17.7%) (Fig. 1).

Of the 688 neonates discharged, 56.7% were discharged alive, 2.5% were discharged with treatment, and 17.3% were referred to the NICU. In contrast, the rest of the 23.5% were discharged with an appointment.

### Neonatal survival

During the study period, a total of 15.1% (*n* = 122) of neonates deaths were observed, making an overall newborn mortality rate of 151 per thousand births. Of the 122 newborn deaths, 84(68.9%) were stillbirths, 38(31.1%) died in the first 72 h (Table 2). The overall incidence of neonates mortality was 1.012 per thousand early neonate hours.

**Table 2:** survival function life table of Amhara regional state referral hospitals, Northern Ethiopia

Time	Beg Total	Fail	Net Lost	Survivor Function	Std Error	[95% Conf. Int.]
1	810	82	0	0.8988	0.0106	0.8759 0.9176
6	728	0	117	0.8988	0.0106	0.8759 0.9176
12	611	0	2	0.8988	0.0106	0.8759 0.9176
20	609	1	1	0.8973	0.0107	0.8742 0.9163
24	607	15	95	0.8751	0.0119	0.8498 0.8964
48	497	6	68	0.8646	0.0125	0.8380 0.8871
50	423	1	0	0.8625	0.0126	0.8357 0.8853
52	422	1	0	0.8605	0.0127	0.8333 0.8835
72	421	16	405	0.8278	0.0146	0.7968 0.8544

### Kaplan-Mair survival analysis

**Fig. 2:**
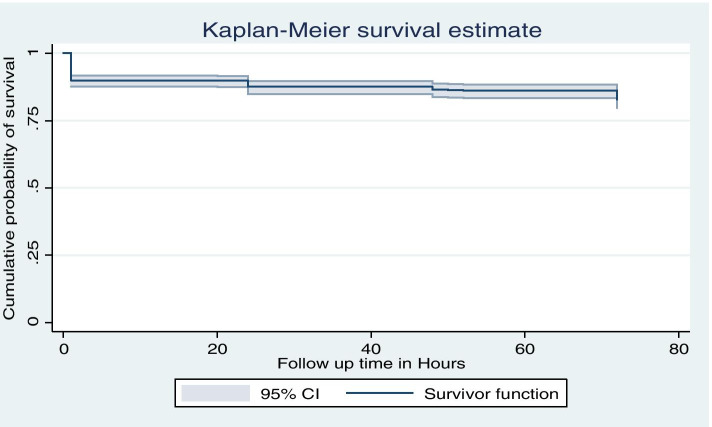
K-M survival estimate of newborns in Amhara Regional state referral hospitals, Northern Ethiopia

**Fig. 3:**
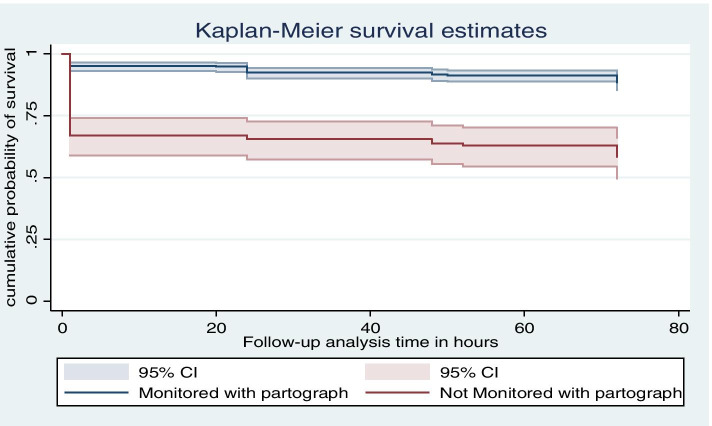
K-M survival estimate of neonates with labour monitored with partograph Amhara Regional state referral hospitals, North-west Ethiopia

There was a higher mortality level in the first 24 h compared to after 24 to 72 h. This finding indicates a substantial reduction in death after the neonates survive the early 24 h of life in the observation period (Fig. 2). Similarly, the curve indicates that neonatal mortality shows substantial decrement among those whose labour was monitored with Partograph (Fig. 3).

### Determinants of neonatal mortality

We included the variables with a *p*-value of less than 0.25 in the Cox proportional hazards regression model’s crude model. Then, after controlling for potential confounders using multivariate Cox proportional hazard regression, the timing of first antenatal care visit, monitoring with partograph, type of admission of the women, and maternal complications within the first 24 h (postpartum haemorrhage, fistula, and obstructed labour) were the variables that determine neonatal survival within the first 72 h of life.

Gestational age at the first antenatal care visit was found to be a risk factor for neonatal mortality. Women who came between 17 and 28 weeks of gestation for the first visit were 1.67 times more likely to lose their child [AHR = 1.67: 95% CI: 1.02, 2.73] than those who started the initial antenatal care visit before 16 weeks of gestation. Mothers not monitored with a partograph during labour were 2.66 times the risk of neonatal mortality [AHR = 2.66:95% CI: 1.70, 4.15] compared to their counterparts. Direct admission was 39% less risk of neonatal death than [AHR = 0.61:95% CI: 0.38, 0.97] those admitted from referral to another health facility.

Maternal complications within 24 h were also a significant risk factor for newborn mortality. Mothers experiencing postpartum haemorrhage were about three times risky for new-borns death [AHR = 2.88; 95% CI:1.69, 4.89], and those who developed fistula in the first 24 h were also about four times risky for new-borns death [AHR = 3.75; 95% CI: 1.23, 11.43]. Obstructed labour was more than twice risky [AHR = 2.14; 95% CI: 1.35, 3.38] for neonatal mortality and less than 1-h maternal first delay in visiting health facility was 39% less risk of neonatal death [AHR =0.61; 95% CI: 0.37, 0.98] (Table 3).

**Table 3:** Bivariate and Multivariate Cox-proportional hazard regression for the first 3 days neonatal mortality in Amhara regional state referral hospitals, Northern Ethiopia

Variables	Outcome		CHR (95%CI)	AHR (95%CI)
	Died	Censored		
**Parity**				
1 child	38(4.69)	292(36.05)	1.0	1.0
2–4 children	48(5.93)	292(36.05)	1.26[.82, 1.93]	1.20[0.74, 1.94]
5 children & above	36(4.44)	(104)12.84	2.25[1.43, 3.56] **	0.92 [0.52, 1.64]
**Gestational age for the first antenatal care visit**				
≤16 weeks	34(4.42)	343(44.55)	1.0	1.0
17 to 28 weeks	62(8.05)	307(39.87)	1.84[1.21, 2.80] *	1.67[1.02, 2.73] *
> 28 weeks	10(1.30)	14(1.82)	4.49[2.22, 9.10] **	1.21[0.51, 2.84]
**Monitored with partograph**				
Yes	63(7.78)	598(73.83)	1.0	1.0
No	59(7.28)	90(11.11)	4.48[3.13, 6.39] **	2.66[1.70, 4.15] **
**Maternal mode of admission**				
Direct admission	44(5.43)	437()	.37[.25, .53] **	0.61[0.38, 0.97] *
Referral	78(9.63)	251(30.99)	1.0	1.0
**Mothers complications within 24 h**				
Abdominal distension	23(2.84)	68(8.40)	2.63[1.63, 4.26] **	1.24[.67, 2.32]
PPH	34(4.20)	38(4.69)	5.79[3.79, 83] **	2.88[1.69, 4.89] **
Fistula	5(0.62)	4(0.49)	6.59[2.64, 16.43] **	3.75[1.23, 11.43] *
No complication	60(7.41)	578(71.36)	1.0	1.0
**Obstructed Labor**				
Yes	74(9.14)	196(24.20)	2.78[1.93, 4.01] **	2.14[1.35, 3.38] *
No	48(5.93)	492(60.74)	1.0	1.0
**Maternal first Delay**				
< 1 h	55(6.79)	560(69.14)	.25[.17, .36] **	0.61[0.37, 0.98] *
≥1 h	67(8.27)	128(15.80)		1.0

**p < 0.001, *p < 0.05, CHR- crude hazard ratio, AHR-adjusted hazard ratio and CI-confidence interval, the bold indicates significant variables

## Discussion

Losing a newborn within the first 3 days of life, during which high neonatal mortality occurred, was shocking for the family and community and is devastating globally. Especially in developing countries, addressing this issue was a complex task for several factors. The study aimed to determine the incidence and determinants in the first 3 days among babies delivered in referral hospitals. In this study, 810 neonates born at the referral hospitals were included during the study period, and male predominance was noted in 53.5% of the study participants. Our study finding is in line with studies carried out in Pakistan (63%) [26], South Africa (57.8%) [27], in India (63.3%), in St Paul’s Hospital Millennium Medical College (61.1%) [28] and University of Gondar hospital (58.3) [22], Ethiopia. Natural selection response to differential survival prospects [29] and cultural and social factors [22] discrepancy between female and male babies. Our study’s causes of neonatal deaths were neonatal jaundice, complications such as birth trauma and congenital anomaly, asphyxia, umbilical sepsis, and neonatal sepsis, which are in line with the causes found in Ghana [30, 31], and Uganda [32].

In our study, 122 neonates were lost within the first 3 days of life, giving an overall neonatal mortality rate of 151/1000 total deaths and a stillbirth rate of 103.7/1000 total births. This figure shows a significant decline from a study conducted in the Tikur Anbesa specialised hospital (225/1000 live births) [33] and (302/1000 live births) [22]. This decline might be due to the impacts of different interventions for the last 6 years. However, it was much higher than the global neonatal mortality rate in 2016 [34], and in studies conducted in Southern Ethiopia [35, 36], Eastern Ethiopia [37], Southwest Ethiopia [38], Sudan [39], Uganda [25], Zambia [40] and Ghana [30].. Our study finding was also much higher than a finding of a systematic review of perinatal mortality rate in Ethiopia that indicated 75/1000 live births at the institutional level, 43/1000 total births with follow up studies, 59.1/1000 total births in the Amhara region, and 29.5/1000 total births among early newborns (up to 7 days) [41]. The variances might be attributed to study designs, health service coverage, socioeconomic factors, and PMR’s definition in other studies. In addition, the higher PMR in this study might be because of the admission of complicated mothers and the consideration of perinatal mortality rate up to 3 days in referral hospitals. This finding implies that the situation of neonatal mortality is still not progressing as anticipated in referral hospitals and strengthen the argument made by the study conducted in Jimma Zone [38] and a previous systematic review [6], which concluded that “health facility delivery had no significant effect on neonatal mortality.” However, this study’s findings should be interpreted vigilantly because of the stillbirth rate reports among admitted term pregnant women in referral hospitals. Possible misclassification of pregnancy outcomes (e.g., severe asphyxia of neonates) might overestimate the actual burden of stillbirth in the study area. Though there might be differences based on some factors, this implies that there is a need to plan tailored and targeted interventions by all stakeholders at different levels.

Regarding the determinants of neonatal mortality within the first 72 h, gestational age at the first antenatal care visit was a risk factor. Women who came between 17 and 28 weeks of gestation for the first visit were 1.67 times more likely to lose their child than those who started the initial antenatal care visit before 16 weeks of pregnancy. This finding is consistent with studies conducted in Tigray regional state [42], Felege Hiwot referral hospital [43], and Gaza-Strip [44]. This result infers that the earlier the start of prenatal care visits, the more the mothers will have time to complete four follow-ups, which will help us a new method of obstetric problems, which suggests the recent WHO recommendation of positive pregnancy experiences [45]. Thus, this study implies that the early start of the antenatal visit and possible consideration of the new WHO recommendation for antenatal care visits in Ethiopian referral hospitals could play vital roles in reducing early newborn deaths.

Maternal complications within 24 h were also a significant risk factor for neonatal mortality. Of these, the experience, postpartum haemorrhage, fistula development within the first 24 h, and obstructed labour were found to be three times, four times, and more than twice risky for neonatal death within the first 72 h of life. Our study’s findings regarding fistula and postpartum haemorrhage as risks for neonatal mortality were unique in this finding. The possible reason for neonatal mortality among mothers facing postpartum haemorrhage and fistula might be intrapartum asphyxia. In cases of maternal complications, the attention of health care providers diverts to saving the mother, and in some cases, neonates would not get adequate care, which leads them to intrapartum asphyxia. However, future research should be conducted to get the exact cause of neonatal mortality in such complications. However, this study’s results, which identified obstructed labour as a risk of neonatal mortality, were similar to studies conducted in Hawassa University hospital, Ethiopia [46], and tertiary hospitals in Tanzania [47]. These findings might be due to asphyxia and prolonged labour-related consequences leading to premature neonatal death.

Moreover, mothers who were not monitored with partograph during labour were nearly three times the risk of neonatal mortality than their counterparts. This result was supported by a study in Addis Ababa [48] and Tigray regional state [42]. This outcome entails that feto-maternal health should be monitored with the start of the active first stage of labour for timely management of prolonged labour, and its consequences will be early identified as prevention and control of early neonatal death.

Furthermore, direct admission was 39% less risk of newborn mortality than those admitted from referral to another health facility. In other words, mothers who require a referral were either suffer from severe obstetric problems or transfer time. We extend the time to receive skilled care. Besides, less than 1 h of maternal first delay to visit health was 39% less risk of neonatal death. This result was similar to a study in Tigray Northern Ethiopia, showing that seeking skilled care at the start of labour was protective for perinatal mortality [42], Uganda [32], and India [49]. This result indicates that the first delay in maternal death also contributes to early neonatal death. We suggest that healthcare providers pay attention to newborns’ care with significant intrapartum asphyxia, including respiratory, temperature, and nutritional support.

Despite the indications of the Ghanian study [30], this study has some inherent limitations. First, though this study was unique in addressing the first 3 days of life with a follow-up study design to determine the risk factors of early neonatal mortality, being only at the tertiary level of care may elevate the actual incidence estimate of premature neonatal death in the region. Second, the study was based on tertiary hospitals and may not show the picture of secondary and primary hospitals, and data were only collected up to 72 h of the life of the newborns. Therefore, cases occurring after 72 h were missed. Third, a mixed-method study design should have been used to identify the issues related to mothers and health care providers’ perceptions of the quality of services provided in the referral hospitals. Further longitudinal studies focusing on early neonatal death should explore health system, maternal, and obstetric factors, especially in the first 3 days.

## Conclusions

We hypothesised high neonatal mortality in tertiary care centres in the first 3 days of life and found that about 1 in 7 newborns died in the first 3 days of life in the tertiary level of care in Northwestern Ethiopia. The leading causes of newborn death were neonatal jaundice, followed by complications like birth trauma, congenital anomalies, and asphyxia.

Moreover, the determinants of neonatal mortality were delay for the first ANC visit, more than 1-h maternal first delay to visit a health facility, and health system-related determinants such as not monitoring labour with, admission by referral were the significant factors. Furthermore, obstetric determinants were mothers’ experience of postpartum haemorrhage, fistula development within the first 24 h, and obstructed labour. Therefore, each hospital in the region must start implementing the WHO recommendation on positive pregnancy experience for addressing the health system and obstetric risk factors of newborn mortality. Additionally, awareness creation and adherence to the recommended level of care are essential.

## Supplementary Information

**Additional file 1.**

## Data Availability

On reasonable request, the corresponding author will provide the datasets generated during the current work.

## Abbreviations

AHR: Adjusted Hazard Ratio
ANC: Antenatal Care
CHR: Crude Hazard Ratio
CI: Confidence Interval
DMH: Debre Markos Hospital
CPAP: continuous positive airway pressure machines
ETB: Ethiopian Birr
FHH: Felege Hiwot Hospital
GA: Gestational Age
SD: Standard Deviation
LMICs: Low- and middle-income countries
NICU: neonatal intensive care unit
RDS: respiratory distress syndrome
UoGH: university of Gondar hospital
WHO: World Health Organization
