# Supplementary material for: Incidence and determinants of neonatal mortality in the first three days of delivery in northwestern Ethiopia: a prospective cohort study
Source: BMC Pregnancy Childbirth. 2021 Sep 23;21:647. doi: 10.1186/s12884-021-04122-8 (PMC8461935; doi:10.1186/s12884-021-04122-8)
Supplement: Supplementary file 1 — Additional file 1. [file 12884_2021_4122_MOESM1_ESM.docx]

# Incidence and determinants of newborn mortality in the first three days of delivery in Northwestern Ethiopia: a prospective cohort study

Mulugeta Dile Worke (MD)^1*^, Afework Tadele Mekonnen (AT)^2^**,** Simachew Kassa Limenh (SK)^3^

*^1^Department of Midwifery, Debre Tabor University, Debre Tabor, Ethiopia, ^2^Population and family health, Jimma University, Jimma, Ethiopia,*

*^3^Department of Midwifery, Bahir Dar University, Bahir Dar, Ethiopia.*

**Consent Form**

The researcher explained the aim of the study. Moreover, to decide at any time if I do not want to participate. Therefore, I assure you that my interest to participate in this study is genuinely from my knowledge.

1. If the client refuses, please go to the next participant.
2. Name of the Hospital:
3. Felege Hiwot referral Hospital
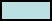

4. UOG teaching Referral Hospital
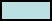

5. Debre Markos Referral Hospital
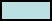

6. Patient Identification No: [_____________]
7. Status of the patient
8. **Non obstructed
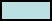
**
9. **Obstructed
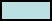
**

| **Part-I: Socio-Demographic Characteristics** | | |
| --- | --- | --- |
| 101 | How much is the mother’s age in years? | [_____] completed Years |
| 102 | Where is the mother Residing? | 1. Urban 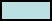 2. Rural 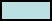 |
| 103 | What is the religion of the mother? | 1. Orthodox 2. Muslim 3. Others (Specify) [______________] |
| 104 | How Much is the mother’s body mass index in kg/m^2^? | [_____] kg/m2 |
| 105 | How much was the Mid upper arm circumference of the mother? | [_____]m |
| 106 | What is the Mothers Ethnicity? | 1. Amhara 2. Agew 3. Other (specify) [______________] |
| 107 | How much is the estimated distance of the mother’s residence from the hospital? | From home [______] Kilometer  From referring HF [______] Kilometer |
| 108 | What is the Mothers Marital Status? If she is not married, go to question No 111. | 1. Unmarried 2. Married 3. Divorced 4. Widowed 5. Separated 6. Cohabit |
| 109 | If the mother is married, what was her age at her first marriage? | [________] Years |
| 110 | What was her age at her first pregnancy? | [________] Years |
| 111 | What is the educational level of the mother? | 1. Unable to read and write 2. Read and write only 3. Primary 4. Secondary 5. More than secondary |
| 112 | If she is married, at what level is the husband of the mother educated? | 1. Unable to write and read 2. Able to read and write only 3. Primary 4. Secondary 5. More than secondary |
| 113 | What is the occupation of the mother? | 1. Housewife 2. Government Employee 3. Self-employed or daily laborer 4. Unemployed 5. Merchant   9.Other (Specify) [_________] |
| 114 | If married, what is the occupation of the mother’s husband? | 1. Farmer 2. Merchant 3. Government Employee 4. self-employed or daily laborer 5. Unemployed   9.Others (Specify) [_________] |
| 115 | How much is the mother the monthly income? | [_________] Ethiopian Birr |

| **Part II. Obstetric Conditions, the incidence of complications and its outcomes** | | |
| --- | --- | --- |
| 201 | Did the mother come to this hospital for the first time?  *[If the answer is no, go to question No 205]* | 1. Yes 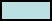 2. No 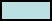 |
| 202 | What was the mode of admission? | 1. Referral  2. Direct admission |
| 203 | If the answer is a referral, what was the source of referral? | 1. Health Post  2. Health Center  3. Hospital  9. Others, specify [_____] |
| 204 | If referred, what was the reason for her referral? | 1. Unable to manage in the other HI  2. Lack of types of equipment in the other HI  3. Lack of supplies in the other HI  4. Lack of trained health professionals in the other HI  5. refusal of the client to be managed in the other HI  9. Others, specify [___________________] |
| 205 | What is the parity level of the mother in number/s? | [____________] |
| 206 | Where was her last delivery? | 1. Health Institution 2. Home |
| 207 | Did the mother have a previous history of abortion?  *[If the answer is no, go to question no 210]* | 1. Yes 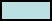  2. No 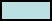 |
| 208 | If the answer is yes, how many abortions? | [___________] Number |
| 209 | If your answer to question No 207 is yes, which type of abortion? | 1. Induced abortion 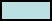  2. Spontaneous abortion 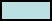 |
| 210 | What is the type of current pregnancy? | 1. Wanted and planned 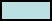  2. Wanted but unplanned 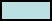  3. Unwanted and unplanned 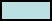 |
| 211 | Have you heard about ANC?  *[If the answer is No, go to question no 215]* | 1. Yes 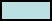  2. No 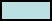 |
| 212 | If you heard about ANC, what was the source of information? | 1. From Media 2. From Health extension workers 3. From community health assistants 4. From friends 5. From health institutions   9. Others, Specify [_______________] |
| 213 | What is the importance of ANC? | 1. For Follow up 2. For vaccination 3. For complication identification 4. For advice and treatment   9. Others, Specify [_____________] |
| 214 | How many times should a mother visit the HF before delivery? | [________] number |
| 215 | Did you have an ANC visit for the current pregnancy?  *[If the answer is no, go to question no 219]* | 1. Yes 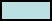  2. No 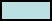 |
| 216 | If you had ANC, what was the time of the pregnancy [GA] at the first visit? | [________] Weeks |
| 217 | If the answer to question No 215 is yes, how many times did she visit ANC? | [________] times |
| 218 | What is the calculated GA at current admission? | [________] Weeks |
| 219 | Does the mother have a history of stillbirth or early neonatal loss? | 1. Yes 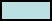  2. No 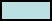 |
| 220 | Did the mother have a previous history of prolonged or obstructed labour? | 1.Yes 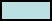  2. No 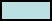 |
| 221 | Did the mother have a previous history of Cesarean Section? | 1. Yes 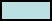  2. No 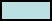 |
| 222 | Which obstetrics complication was detected? | 1. Cephalo pelvic disproportion 2. Malposition 3. Malpresentation 4. Congenital anomaly 5. Pelvic tumour 6. Deep, transverse arrest 7. Contracted pelvis 8. History of a previous fractured pelvis 9. Haemorrhage 10. Hypertensive disorders 11. Infection   9.Others, specify[_____] |
| 223 | If your answer to question No 222 is malpresentation, which type of malpresentation?  *[If the answer is not malposition, go to question No 224]* | 1. Shoulder presentation 2. Breech presentation 3. Face presentation   9.Others [Specify] [____________] |
| 224 | Did the mother come into labourr?  *[If the answer is no, go-to question No 226]* | 1. Yes 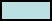  2. No 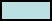 |
| 225 | If the answer to question No 224 is yes, how much was the duration of labour? | [________] Hours |
| 226 | What are the complications detected during labour? | 1. Uterine rupture 2. Haemorrhage 3. Stillbirth 4. Maternal death   9. Others, specify [______________] |
| 227 | Was there a delay in deciding to go to the health Facility?  *[If the answer is no, go to question No 230]* | - - - 1. Yes 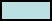       2. No 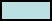 |
| 228 | If your answer to question No 227 is yes, what was the reason for the delay? | 1. Financial Constraints 2. Poor understanding of complications 3. Poor understanding of risk factors 4. Poor understanding of when to seek medical help 5. Previous poor experience of health care 6. Acceptance of maternal death 7. Lack of knowledge where the service will be given 8. Lack of knowledge how to get a transport 9. Culture of the community   9.Others [Specify] [ ______________] |
| 229 | If the answer to Question No 227 is yes, how many hours did it take to decide? | [___________________] hours |
| 230 | Was there a delay in reaching the final place of care after the decision?  *[If your answer is no, go-to question No 233]* | - - - 1. Yes 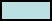       2. No 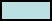 |
| 231 | If your answer to question No 230 is yes, what was the reason? | 1. Long-distance to health facilities 2. Lack of Transport 3. High cost of transportation 4. Poor roads and infrastructure 5. Poor Geographical condition   9.Others [Specify] [ ______________] |
| 232 | If the answer to question No 230 is yes, how many hours she took to reach the hospital? | [___________________] hours |
| 233 | Was there a delay in receiving adequate health care?  *[If the answer is no, go-to question No 236]* | 1. Yes 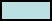 2. No 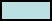 |
| 234 | If your answer to question No 233 is yes, what was the reason? | 1. Lack of medical supplies 2. Inadequately trained HP 3. Poorly motivated medical staff 4. Inadequate referral systems 5. Refusal of medical help 6. Refusal of advice 7. Language barrier   9. Others [Specify] [ ______________] |
| 235 | If your answer to question No 233 is yes, how many hours did it take to receive health care? | [___________________] hours |
| 236 | Was there a delay in making the initial assessment?  *[If your answer is no, go to question No 239]* | 1. Yes 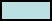 2. No 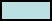 |
| 237 | If your answer to question No 236 is yes, how many hours did it take? | [___________________] hours |
| 238 | If your answer to question No 236 is yes, what was the reason? | 1. Lack of medical supplies  2. Inadequately trained HP  3. Poorly motivated medical staff  4. Refusal of medical help,  5. Refusal of advice  6. Language barrier  9. Others [Specify] [ ______________] |
| 239 | Was there a delay in making a definitive diagnosis?  *[If your answer is no, go-to question No 244]* | 1. Yes 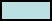 2. No 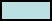 |
| 240 | How many hours did it take to making a definitive diagnosis? | [___________________] hours |
| 241 | If your answer to question No 239 is yes, what was the reason? | 1. Arrived at night 2. Lack of diagnostic materials 3. Prior Incorrect diagnosis 4. Misinterpretation of presenting SMS. 5. Others (specify) [_______________] |
| 242 | What is the time between diagnosis of the primary complication, including obstructed labour and definitive treatment? | [___________] hours |
| 243 | What is the time between diagnosis and attention by health personnel? | [___________] hours |
| 244 | Was the labor monitored by partograph?  *[If the answer is yes, go to question no 247]* | 1. Yes 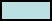 2. No 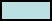 |
| 245 | If monitored, what was the outcome? | 1. Obstructed  2. Not obstructed |
| 246 | If your answer to question No 244 is no, what was the reason? | 1. Inadequately trained HP 2. Poorly motivated medical staff 3. Others, Specify [___________] |
| 247 | What was the mode of delivery? | 1. Spontaneous Vaginal Delivery 2. Assisted vaginal delivery (Instrumental) 3. C/S with tubal ligation 4. C/S without tubal ligation 5. Vaginal birth after CS [VBAC] 6. Hysterectomy 7. Destructive Vaginal delivery   9.Other (specify) [_______________] |
| 248 | Was there a delay in placental delivery?  *[If the answer is no, go to question No 251]* | 1. Yes 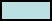  2. No 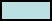 |
| 249 | If the answer is yes, how many hours did it take? | [___________] hours |
| 250 | If the answer to question No 247 is yes, what was the reason? | 1. Inadequately trained health care provider 2. Poorly motivated medical staff 3. Lack of partograph form 4. Work overload in the hospital   9.Others, Specify [_______________] |
| 251 | What occurred to the mother within 24 hours after delivery? | 1. Abdominal distension 2. PPH 3. Fistula 4. Maternal death 5. No complications   9.Others, specify [ _____________] |
| 252 | Was the woman transfused?  *[If your answer is No go to question No 255]* | 1. Yes 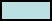  2. No 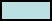 |
| 253 | If your answer to question No 251 is yes, what was the time between the request made for blood transfusion and actual blood transfusion? | [___________] hours |
| 254 | If there was a delay for transfusion, what was the reason? | 1. Lack of blood in the bank 2. Delay of transportation from the bank 3. lack of blood similar to the patient   9. Others Specify [_______________] |
| 255 | What was the weight of the fetus? | [________] grams |
| 256 | What was the APGAR score of the fetus? | 1. [____] First minute and 2. [____] Fifth minute 3. [____] Tenth minute |
| 257 | What was the sex of the baby? | 1. Female 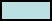 2. Male 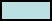 |
| 258 | What was the outcome of the mother? | 1. Alive with no complication  2. Alive with complication  3. Dead |
| 259 | What occurred to the mother within 24-48 hours after delivery?  *[If not discharged within 24 hours]* | 1. Abdominal distension 2. PPH 3. Fistula 4. Maternal death 5. No complications   9.Others, specify [ _____________] |
| 260 | What occurred to the mother within 48-72 hours after delivery?  *[If not discharged within 48 hours]* | 1. Abdominal distension 2. Puerperal sepsis 3. Wound infection 4. PPH 5. UTI 6. Fistula 7. Maternal death 8. No complications 9. Others, specify [______________] |
| 261 | What Occurred to the mother at discharge? | 1. Discharged with good condition 2. Discharged with Appointment 3. Discharged with treatments 4. Dead   9.Others [Specify]____________ |
| 262 | What occurred to the fetus inside the uterus? | 1. Intrauterine fetal death 2. Non-reassuring fetal condition 3. Reassuring fetal condition   9. Others [specify] |
| 263 | What occurred to the newborn during delivery? | 1. Dead 2. Alive with no Complication 3. Alive but complicated   9. Others, Specify [__________] |
| 264 | What occurred to the newborn within 24 hours after delivery? | 1. Asphyxia 2. Neonatal jaundice 3. Umbilical sepsis 4. Neonatal sepsis 5. Neonatal death 6. No complications   9. Others, Specify [____________] |
| 265 | What occurred to the newborn within 24-48 hours after delivery?  *[ if not discharged within 24 hours]* | 1. Asphyxia 2. Neonatal jaundice 3. Umbilical sepsis 4. Neonatal sepsis 5. Neonatal death 6. No complications   9. Others, Specify [____________] |
| 266 | What occurred to the baby within 48-72 hours after delivery?  *[ if not discharged within 24 hours]* | 1. Neonatal jaundice 2. Umbilical sepsis 3. Neonatal death 4. No complications   9. Others, Specify [______________] |
| 267 | What occurred to the baby at discharge? | 1. Discharged alive and well 2. Discharged with treatment 3. Referred to the neonatal ICU 4. Discharged with appointment   9. Others [Specify] ____________ |
| 268 | How many days did she stay in the hospital after admission? | [______] Days |

Thank you for your participation!!!

Name of data collector: ___________________________ Date ___/_______/____sig_________

Name of Supervisor: ___________________________ Date ___/_______/____sig___________
